# Oncolytic potential of Newcastle Disease Virus in feline lymphoma cells: an *in vitro* evaluation

**DOI:** 10.3389/fvets.2025.1484947

**Published:** 2025-06-11

**Authors:** Talita Gabriela Luna Alves, Pedro Luiz Porfirio Xavier, Taismara Kustro Garnica, Arina Lázaro Rochetti, Talal Jamil Qazi, Thiago Henrique Annibale Vendramini, Felipe Haddad, Muhammad Munir, Márcio Antonio Brunetto, Heidge Fukumasu

**Affiliations:** ^1^Laboratory of Comparative and Translational Oncology, Department of Veterinary Medicine, School of Animal Science and Food Engineering, University of São Paulo (USP), Pirassununga, Brazil; ^2^Nutrition and Production Department, School of Veterinary Medicine and Animal Science, Pet Nutrology Research Center, University of São Paulo (USP), Pirassununga, Brazil; ^3^Advanced Cellular Therapy Laboratory, Ribeirao Preto Blood Center Foundation, University of São Paulo (USP), Ribeirao Preto, Brazil; ^4^Division of Biomedical and Life Sciences, Lancaster University, Lancaster, United Kingdom

**Keywords:** cat, virotherapy, oncolytic, Newcastle Disease Virus, new therapies, cancer, *in vitro*, immunotherapy

## Abstract

Lymphoma is one of the most prevalent types of feline cancer. It is characterized as a group of diseases that can affect various organs, such as the gastrointestinal tract, kidneys, thymus, and skin. In feline medicine, the search for alternative treatments is of utmost importance, given the significant number of animals that relapse or are unresponsive to conventional chemotherapy treatment. As an alternative to existing modalities of treatment for a variety of cancers, oncolytic viruses have been studied in the last few years. Those viruses possess a unique ability to target and eliminate cancer cells while simultaneously stimulating an immune response against malignant cells, acting as an immunotherapy. Newcastle Disease Virus (NDV) is an avian paramyxovirus that affects both domestic and wild birds, causing symptoms that range from severe to asymptomatic, depending on the viral strain. Less virulent strains are considered safe for use as a vaccine against Newcastle Disease. In the Oncology field, those strains are also being studied to be used as oncolytic virotherapy for mammals, and several results demonstrate their efficacy *in vitro* and *in vivo*. The present study aimed to explore the oncolytic potential of Newcastle Disease Virus expressing green fluorescent protein (NDV-GFP) in feline lymphoma cells isolated from a FeLV-positive patient with thymic lymphoma. The NDV-GFP infected, replicated, and induced apoptosis in feline lymphoma cells. Therefore, these results provide preliminary evidence of the oncolytic activity of NDV in feline leukemia virus-induced lymphoma.

## 1 Introduction

Lymphoma is a heterogeneous group of diseases that can be classified into various subtypes, including multicentric, gastrointestinal, extranodal, and mediastinal, each with distinct treatment responses and, consequently, different prognoses (1). Mediastinal lymphoma is a type of lymphoma that involves the thymus, mediastinal, and sternal lymph nodes (2). Historically, this disease has been closely associated with feline leukemia virus (FeLV), a retrovirus capable of integrating its genome into the host cell’s native genetic material, establishing a permanent viral reservoir within the cell (3). This may result in somatically acquired insertional mutagenesis, potentially leading to FeLV-induced neoplasms (4).

However, in recent years, the incidence of mediastinal lymphoma in cats has significantly decreased, especially in developed countries, where vaccination and cat population control are more accessible (2, 5–7). Additionally, the introduction of more effective diagnostic tests has helped identify and isolate infected cats, limiting the spread of the virus. This has resulted in a reduction in the number of cats infected with FeLV and, consequently, a decrease in the number of mediastinal lymphoma cases related to the viral infection (8).

Although FeLV is still prevalent in some parts of the world (9–12) and mediastinal lymphoma remains a concern in these regions (13, 14) since cats with FeLV-associated lymphomas tend to have significantly shorter remission and survival times with available chemotherapeutic protocols (1, 13, 15). Recently, a new chemotherapeutic protocol was developed for cats with high-grade multicentric or mediastinal lymphoma in an area endemic for FeLV, known as LOPH (lomustine, vincristine, prednisolone, and doxorubicin). The protocol was well tolerated, and the Median survival time (MST) was better than similar studies with other protocols (16).

Nevertheless, new treatment strategies are still necessary for FeLV-positive cats with lymphoma since the responses to treatment can vary significantly between individual cats, with some achieving long remissions and others experiencing rapid relapses (15). Otherwise, traditional treatments also carry risks of side effects and toxicity (17). Lastly, the growing concern of drug resistance underlines the importance of developing new treatment options since the patient can stop responding to a treatment that once responded well (18). Therefore, ongoing research and the development of novel therapies are essential to improve outcomes for cats with lymphoma, enhancing both their survival and quality of life. As veterinary medicine advances, novel immunotherapies offer promising alternatives that may target tumor cells more precisely.

The use of oncolytic viruses is a therapeutic approach that has gained prominence in recent years, as these agents can directly induce oncolysis and have stimulatory effects on the immune system (19, 20). These viruses can selectively replicate in and destroy tumor cells (21). Most viruses chosen for use in this type of treatment are attenuated strains that can infect and replicate in the chosen species without causing significant side effects (22). The discovery of oncolytic viruses happened by chance, as it was observed that cancer patients infected with certain types of viruses experienced temporary remission of their disease (23). In recent years, clinical trials have demonstrated their efficacy, which can vary according to the type of virus and the delivery method that is being used (22).

In Veterinary Medicine, several viral species are being tested *in vitro* and *in vivo* for their oncolytic effects, such as vesicular stomatitis virus (24–27), adenovirus (28–31), Sendai virus (32), measles virus (33, 34), vaccinia virus (35–43), myxoma virus (44, 45), herpes virus (46, 47), reovirus (48–52), canine distemper virus (53–56) and zika virus (57). The Newcastle Disease Virus (NDV) presented oncolytic activity in various types of cancer, including canine primary and metastatic melanoma (58), canine mammary carcinoma (59–61) and several intracranial tumors (62). For human (63, 64) and canine lymphoma cells (63), NDV infected selectively the tumor cells and cell death involved apoptosis analyzed by flow-cytometry. However, more preclinical studies are necessary to explore NDV’s application in different types of lymphoma, including feline lymphomas.

The NDV is an enveloped, non-segmented avian virus with negative-sense RNA belonging to the genus Orthoavulavirus and the family Paramyxoviridae (APMV-1) (65, 66). It exhibits variable virulence, with the more virulent strains causing Newcastle disease (67). NDV strains can be classified as lentogenic, mesogenic, and velogenic according to the symptoms of NDV in chickens (no disease, moderate-to-severe disease, and severe disease with high mortality respectively).

In the poultry industry, lentogenic strains are used globally as live-attenuated vaccines (68, 69); for mammals, NDV has been studied to be used as a vaccine vector (69). There are rare documentations of NDV infections in humans, and those are mostly reported among poultry workers with self-resolving conjunctivitis and no transmission of the virus among humans (70). In cats (and dogs), a study has evaluated NDV as a virus-vectored rabies vaccine, and it has shown its safety even with repeated inoculation with high dosages with no severe clinical signs observed (71).

Hence, we assessed whether NDV is oncolytic for feline lymphoma cells by evaluating the antitumor activity of a recombinant lentogenic (low virulence) strain expressing green fluorescent protein (NDV-GFP) in a feline lymphoma cell line isolated from a cat with thymic lymphoma and positive for FeLV (FeLV3281).

## 2 Materials and methods

### 2.1 Cell line

The cell line (FeLV3281) was previously isolated, characterized (72) and acquired from the Cell Bank Riken (Japan). These cells originated from a cat (*Felis catus*) with thymic lymphoma and were positive for the Feline Leukemia Virus subtype A. The cells were maintained in 75 cm^2^ flasks at 37°C and 5% CO_2_ in Gibco Roswell Park Memorial Institute (RPMI) 1,640 medium supplemented with 10% fetal bovine serum (FBS) and 1% Pen-Strep antibiotic. The passage of cells was performed every 3 days, and experiments were conducted using cells between passages 10 and 20. The cells were observed daily using optical microscopy (Axio Vert A1, Zeiss, Jena, Germany). All reagents used for cell culture were purchased from Thermo Fisher Scientific (California, United States) unless otherwise specified.

### 2.2 Virus titration and morphological analysis

The virus used in this study was a genetically modified LaSota strain expressing GFP (NDV-GFP) (73) and was kindly provided by Dr. Muhammad Munir (Lancaster University, United Kingdom). The virus titer was obtained by calculating tissue culture infectious dose 50% method per milliliter (TCID_50_/mL) using the Reed and Muench method (74). Briefly, FeLV3281 cells were seeded at 4.5 × 10^4^ cells/well in 96-well plates containing RPMI 1640 medium supplemented with 2% FBS and 1% Pen-Strep antibiotic. After being plated, the cells were immediately exposed to different concentrations of the virus (10^−1^ to 10^−11^) in octuplicate, and the cytopathic effects were monitored for 5 days as is commonly determined by the Reed and Muench method. The cytopathic effects evaluated were syncytia formation, cell lysis, vacuolization, clumping, and cell death. Images of the cells were captured in both bright-field, to evaluate the cytopathic effects, and fluorescence field, to evaluate the expression of GFP, using ZEISS—Axio Vert A1 with an Axio Can 503 camera attached using a 520 nm wavelength filter for green color (ZEISS, Germany) every 24 h for 120 h.

### 2.3 NDV cytotoxicity assay

The FeLV3281 cells were added to 96-well plates at a density of 1 × 10^4^ cells/well in RPMI 1640 medium supplemented with 2% FBS, 1% Pen-Strep antibiotic, 2% Gluta-MAX, and 1% HEPES. Then, cells were immediately exposed to NDV-GFP that had undergone serial dilutions in pure RPMI 1640 with a Multiplicity of Infection (MOI) of 1 × 10^0^, 2 × 10^−1^, 4 × 10^−2^, 8 × 10^−3^, 1.6 × 10^−3^, 3.2 × 10^−4^, 6.4 × 10^−5^, and 1.28 × 10^−5^ based on the TCID_50_/mL of 3.433 × 10^6^. MOI was estimated by assuming a correlation between TCID₅₀/mL and the number of infectious particles per cell. The assay was conducted in triplicate, having 3 plates with the same conditions. The plates were incubated with the virus for 24 h. The cells were observed under bright-field and fluorescence conditions, and images of each dilution were captured. Differences between wells treated with different virus dilutions and cells not infected with the virus were compared by visualizing the wells under the microscope. To analyze the cytotoxicity of NDV-GFP, the half-maximal inhibitory concentration (IC_50_) of the virus was determined using the CellTiter-Blue® reagent (Promega, United States). At the end of the viral treatment, 20 μL of CellTiter-Blue® was added to all wells, and the cells were incubated at 37°C for more than 24 h. The plates were analyzed using a spectrophotometer at wavelengths of 540 nm and 630 nm (LMR 96, Loccus, Brazil), and cell viability was measured in terms of absorbance. The IC_50_ was determined using GraphPad Prism software (version 8.0; United States), using a nonlinear regression method with dose–response inhibition parameters.

### 2.4 NDV cell death assay

The FeLV3281 cells were added to T25 cm^2^ flasks at a concentration of 1 × 10^6^ cells per flask in RPMI 1640 medium supplemented with 2% FBS, 1% Pen-Strep antibiotic, 2% glutaMAX, and 1% HEPES. Subsequently, the cells were immediately exposed to the IC_50_ calculated in the cytotoxicity assay. The assay was conducted in triplicate, having 3 wells with the same conditions. The flasks were maintained in a CO_2_ incubator at 37°C for 24 h. The cells were observed under bright-field and fluorescent conditions, and images in each field were captured. To analyze whether the observed cytopathic effects were triggered by an increase in the number of cell death, a flow cytometry assay using propidium iodide (PI) was conducted following a protocol from Riccardi et al. (75). Briefly, the cells were centrifuged at 1,200 rpm for 5 min, and the supernatant was discarded, and the cells were resuspended in 500 μL of PBS. Cells were fixed in 4.5 mL of 70% chilled ethanol and stored at −20°C for at least 24 h. The cells were centrifuged at 1,800 rpm for 5 min, the supernatant was discarded, and the cells were washed in 5 mL PBS and centrifuged at 1,800 rpm for 5 min. The cells were then resuspended in 1 mL of PI staining solution (200 μg in 10 mL PBS and 2 mg DNAse-free RNAse) and incubated for half an hour at room temperature. It is important to note that the use of propidium iodide (PI) staining alone assesses membrane integrity and does not allow us to distinguish between late apoptosis and necrosis. 20,000 events were captured using a flow cytometer (S3e Cell Sorter, BIO-RAD, Hercules, CA, United States) with a 488-nm laser line for excitation, measuring red fluorescence (4,600 nm) and side scatter. The gating strategy consisted of an initial exclusion of debris based on a side scatter area (SSC-A) vs. forward scatter area (FSC-A) plot. Singlet cells were then selected using a FSC-A versus FL2-A (PI fluorescence) plot to exclude doublets and aggregates. Finally, PI fluorescence was analyzed using a histogram (count vs. FL2-A). Statistical analyses were performed by the unpaired t-test.

## 3 Results

### 3.1 NDV-GFP infects and replicates in feline lymphoma cells

**Figure 1:**
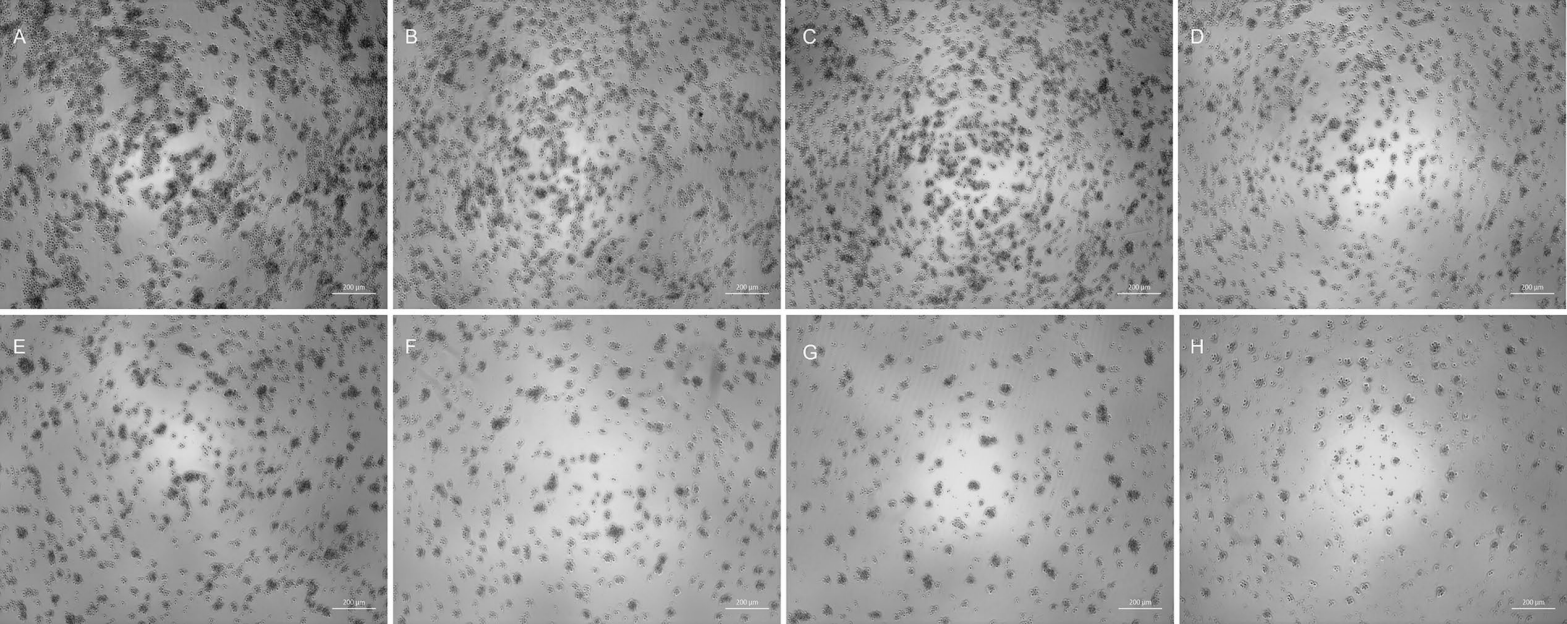
Viral titration by the Reed-Muench method with analysis of cytopathic effects in bright-field 120 h post-infection. Control well **(A)** and wells infected with viral dilution of 10^−11^ **(B)**, 10^−8^ **(C)**, 10^−5^ **(D)**, 10^−4^ **(E)**, 10^−3^ **(F)**, 10^−2^ **(G)**, and 10^−1^ **(H)**. Arrows **(H)** indicate cellular debris. As the virus concentration increased, the well confluence decreased, leading to an increased number of cellular debris observed in dilutions 10^−1^ and 10^−2^. Although GFP expression can only be observed up to 72 h post-infection, the evaluation of cytopathic effects is performed up to 120 h post-infection according to the Reed-Muench method. 50x magnification.

**Figure 2:**
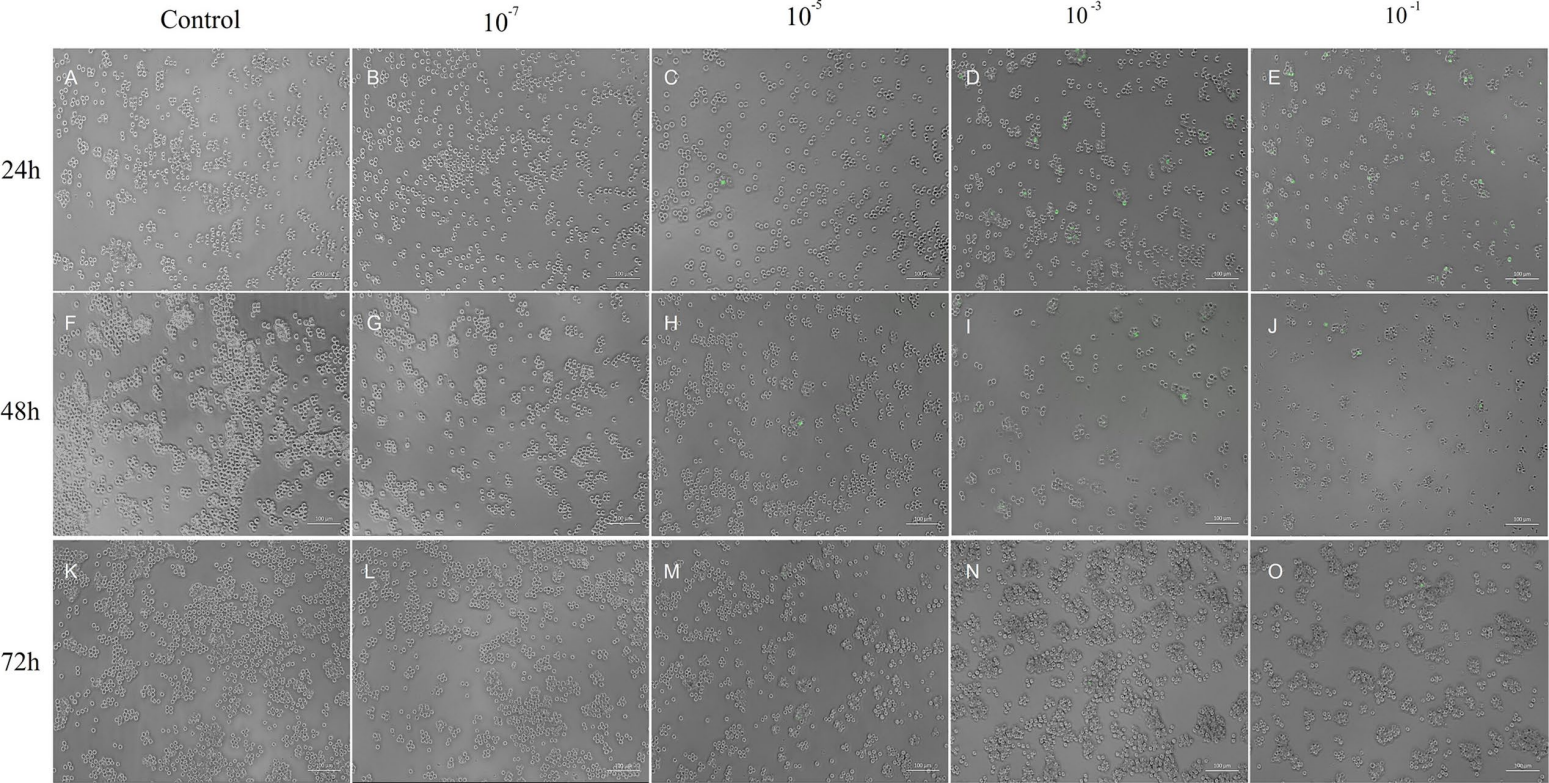
Viral titration assay. Besides control (A, F and K), it is possible to observe FeLV3281 cells that did not express GFP at the concentration of 10^−7^ (B, G and L). At a concentration of 10^−5^ (C, H and M), 10^−3^ (D, I and N) and 10^−1^ (E, J and O), FeLV3281 cells express GFP. The cells were evaluated using bright-field and fluorescent microscopy at 24 **(A–E)**, 48 **(F–J)**, and 72 h **(K–O)** post-infection (p.i.), regarding GFP expression assessment and evaluation of cytopathic effects. Higher GFP expression was observed within the first 24 h of the assay. When comparing control and treatment wells, it is suggested by the observation of cells under microscopy that the confluence is lower in the treatment wells. 100x magnification.

The NDV-GFP reduced the feline lymphoma cells dose-dependently (Figure 1). The titter obtained for NDV-GFP in FeLV3281 cells was 3.433 × 10^6^ tissue culture infectious dose per milliliter (TCID_50_/mL) after 120 h of observation of the cytopathic effects. No syncytia were observed. Although it was observed cell lysis and cell death (Figure 1). The detection of GFP indicated the virus was viable and was able to infect and replicate in the feline lymphoma cells as observed from 24 h post-infection (p.i.) in all wells from the first to the fifth dilution (10^−1^ to 10^−5^) and persisted up to 72 h p.i., albeit with lower intensity over the days (Figure 2). The GFP was not detected from the 10^−7^ dilution along (Figure 2).

### 3.2 NDV-GFP is cytotoxic and induces cell death in lymphoma cells

**Figure 3:**
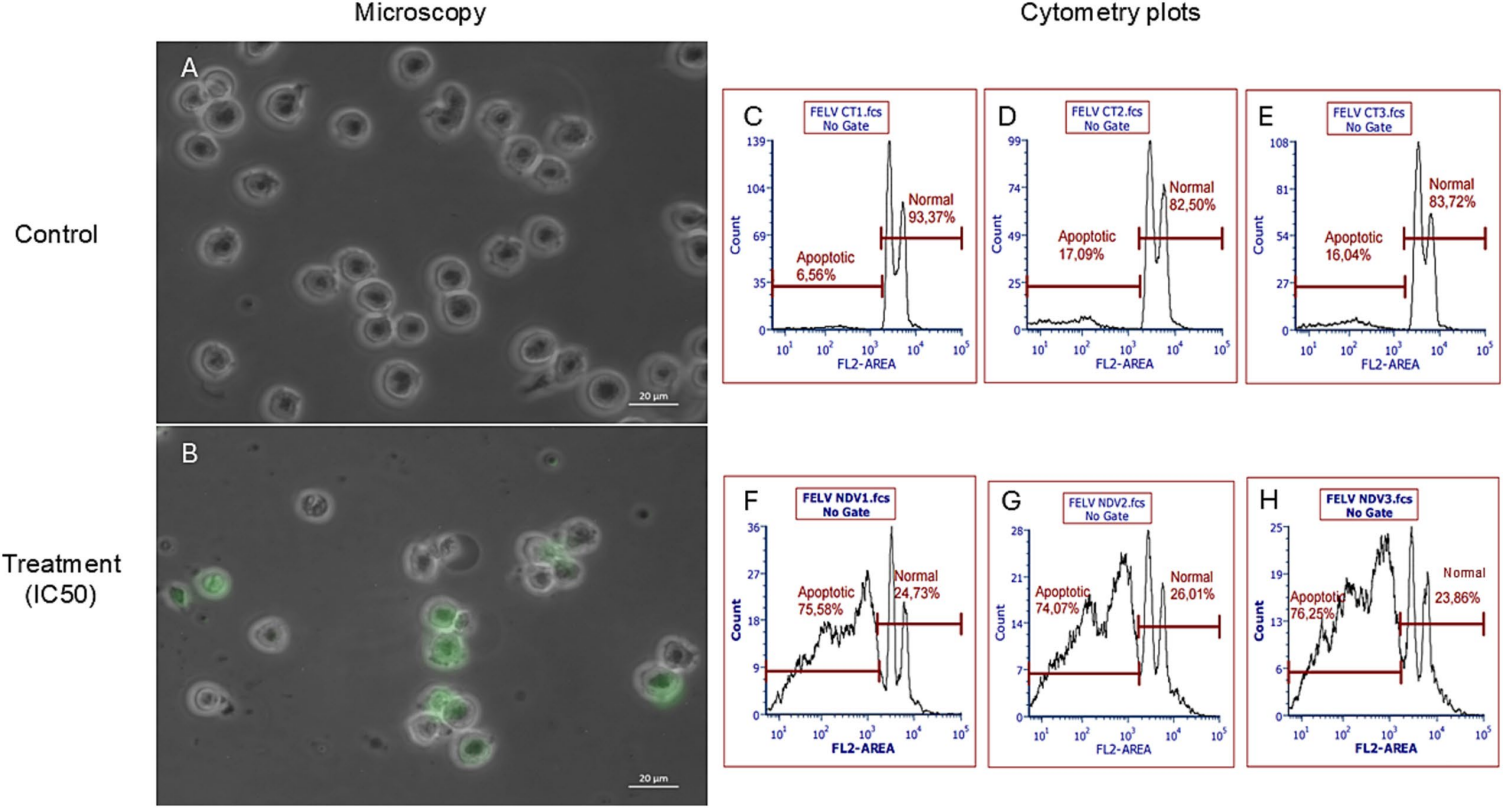
Evaluation of the effects of NDV-GFP on feline lymphoma cells (FeLV3281 cell line). A decrease in the number of viable cells as well as expression of GFP was observed in cells treated with the IC_50_ **(B)**, but not in untreated cells **(A)**. Bright-field and fluorescence images were superimposed **(A,B)** to further highlight GFP expression. The percentage of apoptotic cells and viable cells in non-treated **(C–E)** and treated **(F–H)** cells was compared by flow cytometry analysis. “FL2 area” is the measure of the total fluorescence captured in the FL2 channel.

**Figure 4:**
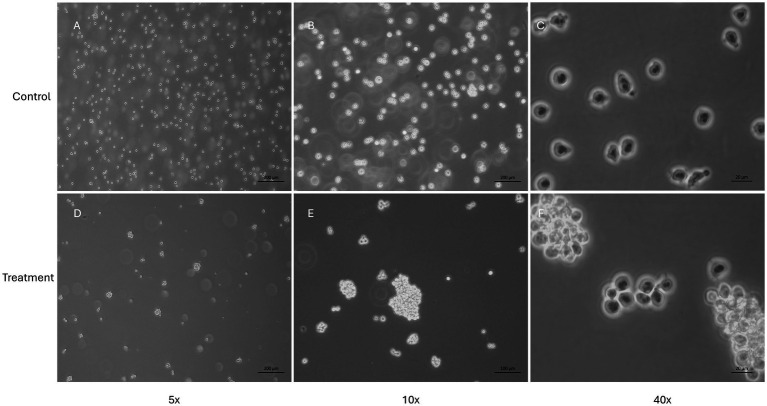
Evaluation of feline lymphoma cells (FeLV3281 cell line) treated with the IC_50_ of NDV-GFP. This figure presents the morphology of cells unexposed to NDV-GFP **(A–C)** and cells treated with the IC_50_ concentration of NDV-GFP **(D–F)**. In virus-treated wells, a noticeable reduction in cell confluence is observed, along with the formation of distinct cell clumps, suggesting cytopathic effects induced by the oncolytic virus. In contrast, control cells maintain a typical morphology, with no cell clumps and a higher confluence.

The NDV-GFP is oncolytic for FeLV3281 cells, as demonstrated by a dose-dependent effect on cell viability with an IC_50_ of MOI = 3.201 × 10^−1^ ± 0.04 (Supplementary Table S1). When compared to untreated cells (Figure 3A), there was observed a reduction of cell number in cells treated with the IC50 of the NDV-GFP. Besides, GFP was detected in cells treated with the IC50 of the virus (Figure 3B), unlike untreated cells (Figure 3A). In cell death analysis, it was also observed a significantly higher occurrence of cell death was observed in cells treated with the IC_50_ (61.95% ± 1.68%) compared to untreated cells (1.80% ± 0.39%, *p* < 0.0002; Figures 3C–H; Supplementary Table S2). Cell clumping was also observed (Figure 4).

## 4 Discussion

There is an urgent need for the development of new candidate therapies for feline lymphoma due to its frequency and the drawbacks of conventional chemotherapy, such as tumor relapse and the possibility of side effects. Here, we demonstrated the oncolytic effect of NDV-GFP in the feline lymphoma cell line FeLV328, as shown by its ability to infect, replicate, and induce morphological changes, culminating in cell death in cancer cells. Additional evidence demonstrated these effects as dose-dependent in the lymphoma cells.

In this study, we used a recombinant lentogenic La Sota variant of NDV-GFP that allowed the detection by fluorescent microscopy of the GFP-expressing cancer cells after virus exposure, assuring that this NDV-GFP infected and replicated in cancer cells. This virus was initially created to determine the tissue tropism of non-virulent NDV in chicken embryos (73). It became a good tool for researching new cancer therapies based on NDV since this virus could be genetically modified to express other proteins or ncRNAs of interest by reverse genetics. Another advantage of the lentogenic NDV-GFP for cancer therapy is the fact that it can be considered safe in non-natural hosts like mammals since it typically results in mild clinical signs, such as conjunctivitis and flu-like symptoms, mostly when exposed to more virulent strains or higher doses of the virus (76–78). The absence of syncytia formation in cancer cells after NDV-GFP infection supports this virus as lentogenic, non-virulent, and non-lytic, as we have already demonstrated in canine cancer cells (59).

Although the US-FDA has not approved any NDV therapy for any condition so far (April 2025), several clinical trials with NDV were performed in humans (more information found in: https://www.cancer.gov/about-cancer/treatment/cam/hp/ndv-pdq). Most human clinical trials of NDV for cancer treatment have been conducted as phase I or II clinical trials typically employing a lytic strain, an oncolysate, or vaccines derived from virus-infected tumor cells. There is only one ongoing phase I clinical trial by AstraZeneca (ID: D7880C00001) of a recombinant NDV expressing Interleukin-12 (MEDI9253) registered on the astrazenecaclinicaltrials.com website. This study was completed on May 31, 2024. On the other hand, to the best of our knowledge, this is the first description of the oncolytic effect of NDV in feline tumors.

An important limitation of this study is the absence of an evaluation of NDV’s cytotoxic effects on non-cancerous feline cells, such as peripheral blood mononuclear cells (PBMCs) or fibroblasts. Assessing these cell types is essential to determine the virus’s selectivity toward cancer cells and to characterize its safety profile. Therefore, it is highly recommended that future studies include such analyses before advancing to preclinical and clinical investigations. Nevertheless, previous studies have already demonstrated that NDV is selective for dog cancer cells (59), as well as being safe for use in dogs (60, 61), and at least one study has assessed the safety profile of NDV in cats (71), reporting no significant adverse effects, which supports its potential as a safe oncolytic agent in veterinary oncology.

We have performed cytotoxicity assays using PBMCs treated with serial dilutions of NDV-GFP (1 × 10^0^, 2 × 10^−1^, 4 × 10^−2^, 8 × 10^−3^, 1.6 × 10^−3^, 3.2 × 10^−4^, 6.4 × 10^−5^, and 1.28 × 10^−5^ MOI, based on the TCID_50_/mL of 3.433 × 10^6^ – the same methodology used for the lymphoma cell line, FeLV3281) and incubated for 24 h, but the results were inconclusive. In this assay, we have also used the CellTiter-Blue® with the porpuse of analyzing cell viability. CellTiter-Blue® have resazurin as the main reagent. Resazurin is a blue and non-fluorescent dye. In viable cells, it is reduced by cellular metabolic activity into resorufin, which is pink and fluorescent, resorufin. This reduction did not happen with PBMCs, even in control cells (cells that were not treated with the virus). All dilutions (and control wells) remained with the same blue dye, even after 24 h of exposition to the reagent. We hypothesize that PBMCs do not metabolize CellTiter-Blue®, because they are not metabolic active cells and need to be activated with mitogens, such as Concanavalin A (Con A). Therefore, to conduct cell viability assays with CellTiter-Blue®, it is necessary to discover the concentration of Con A that can induce PBMCs to a metabolic active state and, hence, induce the reduction of resazurin to resorufin in viable cells.

Another fact that it would be important to evaluate in the future is how immunosuppressed animals would respond to therapies based on recombinant non-lytic oncolytic viruses, with emphasis on FeLV-positive cats with lymphoma. It is known that innovative therapies need to be developed to improve the survival rate and the quality of life of those animals that are often diminished because of the interaction of both diseases (15, 79–81). Besides the poor prognosis, FeLV is still a disease that has a high prevalence in some countries (9, 13, 14, 82, 83). Therefore, treating cats with FeLV-associated lymphoma is a subject that needs to be addressed with the necessary degree of urgency.

Finally, it is important to note that our study was conducted using only a single FeLV-positive feline lymphoma cell line (FeLV3281). While our findings offer valuable preliminary evidence of NDV-GFP’s oncolytic potential in this specific model, they may not fully reflect the behavior of other feline lymphoma subtypes or FeLV-negative cases. Future studies should include additional cell lines encompassing both FeLV-associated and non-FeLV-associated models to further validate and extend these observations. We also acknowledge that the exclusive use of PI staining has limitations, as it does not differentiate between late apoptotic and necrotic cells. Nevertheless, the dose-dependent cytotoxic effects observed, along with supportive morphological and cell viability data, provide robust evidence that NDV-GFP predominantly induces apoptosis in the FeLV-positive feline lymphoma cell line. In future studies, we plan to further delineate the cell death pathways by incorporating additional markers, such as caspase activity assays.

In conclusion, these preliminary results provide support for the continuation of *in vitro* studies using oncolytic NDV candidates for the treatment of feline lymphoma, which is an urgent necessity due to the limitations of currently adopted treatment modalities, such as chemotherapy. Future work will extend our investigation into *in vivo* preclinical models to further assess the safety and therapeutic efficacy of NDV-GFP. Specifically, we plan to develop xenograft models in immunodeficient mice using FeLV-positive feline lymphoma cells to simulate the tumor microenvironment and monitor treatment response in a controlled setting. Additionally, primary tumor cultures derived from FeLV-positive cats will be explored, as these models are likely to reflect better the heterogeneity and complexity of naturally occurring feline lymphoma. These complementary approaches will help bridge our *in vitro* findings with clinical relevance and guide the design of subsequent clinical studies.

## Data Availability

The original contributions presented in the study are included in the article/Supplementary material, further inquiries can be directed to the corresponding author.
